# *Streptococcus suis* 5’-nucleotidases contribute to adenosine-mediated immune evasion and virulence in a mouse model

**DOI:** 10.1080/21505594.2024.2401963

**Published:** 2024-09-16

**Authors:** Simin Deng, Haojie Li, Chang Zhou, Jingyan Fan, Fuxin Zhu, Gexuan Jin, Jiali Xu, Jing Xia, Jing Wang, Zheng Nie, Rui Zhou, Houhui Song, Changyong Cheng

**Affiliations:** a Key Laboratory of Applied Technology on Green-Eco-Healthy Animal Husbandry of Zhejiang Province, Zhejiang Provincial Engineering Laboratory for Animal Health Inspection & Internet Technology, Zhejiang International Science and Technology Cooperation Base for Veterinary Medicine and Health Management, China-Australia Joint Laboratory for Animal Health Big Data Analytics, College of Veterinary Medicine of Zhejiang A&F University, Hangzhou, Zhejiang, P.R. China; b College of Veterinary Medicine, Huazhong Agricultural University, Wuhan, Hubei, P.R. China

**Keywords:** *Streptococcus suis*, 5’-nucleotidases, virulence factors, enzymatic activity, adenosine pathway

## Abstract

*Streptococcus suis* (*S. suis*) is an important swine bacterial pathogen and causes human infections, leading to a wide range of diseases. However, the role of 5’-nucleotidases in its virulence remains to be fully elucidated. Herein, we identified four cell wall-anchored 5’-nucleotidases (Snts) within *S. suis*, named SntA, SntB, SntC, and SntD, each displaying similar domains yet exhibiting low sequence homology. The malachite green reagent and HPLC assays demonstrated that these recombinant enzymes are capable of hydrolysing ATP, ADP, and AMP into adenosine (Ado), with the hierarchy of catalytic efficiency being SntC>SntB>SntA>SntD. Moreover, comprehensive enzymatic activity assays illustrated slight variances in substrate specificity, pH tolerance, and metal ion requirements, yet highlighted a conserved substrate-binding pocket, His–Asp catalytic dyad, metal, and phosphate-binding sites across Snts, with the exception of SntA. Through bactericidal assays and murine infection assays involving in site-mutagenesis strains, it was demonstrated that SntB and SntC collaboratively enhance bacterial survivability within whole blood and polymorphonuclear leukocytes (PMNs) *via* the Ado-A2aR pathway *in vitro*, and within murine blood and organs *in vivo*. This suggests a direct correlation between enzymatic activity and enhancement of bacterial survival and virulence. Collectively, *S. suis* 5’-nucleotidases additively contribute to the generation of adenosine, influencing susceptibility within blood and PMNs, and enhancing survival within blood and organs *in vivo*. This elucidation of their integral functions in the pathogenic process of *S. suis* not only enhances our comprehension of bacterial virulence mechanisms, but also illuminates new avenues for therapeutic intervention aimed at curbing *S. suis* infections.

## Introduction

The innate immune system is a highly regulated and interconnected cellular network that collaboratively maintains and restores host homoeostasis while protecting against infection by diverse microorganisms, including bacteria [1]. The key constituents of the innate immune system in defence against invading microorganisms include physical barriers, antimicrobial peptides, complement system factors, antibodies, collectins, and ficolins as freely secreted antimicrobial proteins found in the blood, as well as leukocytes such as polymorphonuclear leukocytes (PMNs) and mononuclear phagocytes [2,3]. However, microbial pathogens have evolved mechanisms to evade, suppress, and manipulate innate immune defences. These pathogens often target the initial points of microbial recognition and can influence host cell signalling, production of pro-inflammatory cytokines, and trafficking and secretion of proteins. The balance between immune activation and suppression is intricately regulated, allowing an optimal host response against infection while simultaneously limiting collateral immune-mediated damage to host tissues [4].

Adenosine (Ado), a key biomolecule implicated in modulating the host response to infections and tissue damage, is a particularly potent immunosuppressive signal. Ado is produced from ATP, ADP, and AMP, catabolized by 5’-nucleotidases, which are distributed across all life forms and various cellular locations [5,6]. Ado not only prevents over-exuberant inflammation to protect the host during acute infections but might also harm the host by dampening the protective antimicrobial response; therefore, the balance between ATP/ADP/AMP and Ado concentration is important in immune homoeostasis [4,7]. Thus, 5’-nucleotidases that convert ATP, ADP, and AMP to Ado are involved in cell-matrix or cell-cell interactions and transmembrane signalling and play important roles in immune and inflammatory responses. 5’-Nucleotidases can be classified based on their mechanism of hydrolysis and the type of molecule used as the initial acceptor of the substrate phosphoryl group: an activated water molecule for the type I catalytic mechanism or a nucleophilic amino acid residue for the type II catalytic mechanism [8]. Microbial 5’-nucleotidases that utilize the type I catalytic mechanism have been identified and characterized in Gram-positive (*Staphylococcus*, *Streptococcus*, and *Bacillus species*) and Gram-negative (*Vibrio*, *Shewanella*, and *Legionella species*) bacteria. These 5’-nucleotidases show low but significant sequence identity with mammalian ecto-5’-nucleotidase CD73 [5,9], indicating their common ancestry and similar structures [10,11]. The N-terminal domain of these proteins, which contains a dimetal centre and the catalytic dyad Asp-His in the active site, is characteristic of the calcineurin-like phosphoesterase superfamily (PF00149.28) of phosphatases, while the C-terminal domain is characteristic of the 5’-NT superfamily (PF02872.18) [6]. In Gram-positive bacteria, 5’-nucleotidases increase Ado levels, thereby helping these bacteria compromise the host’s immune defences and survive in host tissues during infection [12], and in some cases also convert dAMP to dAo to trigger caspase-3-mediated death of immune cells [13–15]. *Streptococcus suis* (*S. suis*) is an important Gram-positive porcine pathogen and zoonotic agent that causes septicaemia, meningitis, arthritis, and many other diseases [16–18]. Since the first reported cases of human *S. suis* infection in Denmark in 1968, more than 1600 cases have been reported worldwide [19]. Among *S. suis* 29 serotypes, serotype 2 (SS2) is the most virulent and prevalent one in swine and humans worldwide [20]. SS2 causes two large-scale outbreaks that occurred in 1998 and 2005 in China resulting in 229 infections and 52 deaths [21], particularly because of the cases presented with streptococcal toxic shock-like syndrome (STSLS). Bacterial pathogens evade host innate immune defences and maintain a high dose in blood causing bacteraemia and septicaemia. During these processes, the precise role and mechanism of 5’-nucleotidases in the virulence of *S. suis* remains to be elucidated.

In this study, we identified and characterized four Streptococcal 5’-nucleotidases (Snts) in *S. suis* that are capable of converting ATP, ADP, and AMP into the immunosuppressive molecule Ado to varying extents. Moreover, it was demonstrated that Ado generation capacity is positively correlated with enhanced bacterial survival in whole blood and PMNs, colonization within the blood and specific organs *in vivo*, and lethality rate in infected mice. These insights contribute to the expanding knowledge of the multifaceted functions of 5’-nucleotidases, underscoring their significant impact on bacterial pathogenicity.

## Material and methods

### Bacterial strains and culture conditions

*S. suis* serotype 2 strain SC-19 (GenBank accession number: CP020863.1) used in this study was isolated from a sick pig during an epidemic outbreak in the Sichuan Province of China in 2005 [22]. The *S. suis* strains were grown in brain heart infusion (BHI; Oxoid, United Kingdom) broth or on BHI agar plates supplemented with 5% newborn bovine serum (Tianhang, China) at 37°C. The bacterial strains and plasmids, primers used in the present study are listed in Table 1 and S1, respectively.

**Table 1.** Bacterial strains and plasmids used in this study.

Strains and plasmids	Description and sequence (5’ → 3’)	Source, reference, and products
***E. coli*****strains**		
DH5α	Host for cloning vector	Tsingke
Rosetta (DE3)	Host for expression vector	Tsingke
***S. suis s*****trains**		
SC-19	*S. suis* serotype 2, the wild-type	[22]
*ΔsntA*	The *sntA* (B9H01_10070) single gene-deletion strain	This study
*ΔsntB*	The *sntB* (B9H01_04925) single gene-deletion strain	This study
*ΔsntC*	The *sntC* (B9H01_07430) single gene-deletion strain	This study
*ΔsntD*	The *sntD* (B9H01_10185) single gene-deletion strain	This study
*Δsnts*	The *sntA, sntB, sntC* and *sntD* four gene-deletion strain	This study
C*ΔsntA*	The complementary strain of *ΔsntA*; *Spc*^*r*^	This study
C*ΔsntB*	The complementary strain of *ΔsntB*; *Spc*^*r*^	This study
C*ΔsntC*	The complementary strain of *ΔsntC*; *Spc*^*r*^	This study
C*ΔsntD*	The complementary strain of *ΔsntD*; *Spc*^*r*^	This study
*sntA*_H209A_	Site-mutagenesis strain with His-209 in *sntA* to Ala (cac→gca)	This study
*sntB*_H161A_	Site-mutagenesis strain with His-161 in *sntB* to Ala (cat→gca)	This study
*sntC*_H132A_	Site-mutagenesis strain with His-132 in *sntC* to Ala (cat→gca)	This study
*sntD*_H82A_	Site-mutagenesis strain with His-82 in *sntD* to Ala (cat→gca)	This study
snts*	Four site-mutagenesis strain with His-209 in *sntA*, His-161 in *sntB*, His-132 in *sntC*, His-82 in *sntD* to Ala (cat→gca)	This study
**Plasmids**		
pET30a*_sntA*	Expression vector to produce His_SntA protein; lacZ, *Kana*^*r*^	This study
pET30a*_sntB*	Expression vector to produce His_SntB protein; lacZ, *Kana*^*r*^	This study
pET30a*_sntC*	Expression vector to produce His_SntC protein; lacZ, *Kana*^*r*^	This study
pET30a*_sntD*	Expression vector to produce His_SntD protein; lacZ, *Kana*^*r*^	This study
pET30a*_sntA*_H209A_	Expression vector to produce His_SntA_H209A_ protein; lacZ, *Kana*^*r*^	This study
pET30a*_sntB*_H161A_	Expression vector to produce His_SntB_H161A_ protein; lacZ, *Kana*^*r*^	This study
pET30a*_sntC*_H132A_	Expression vector to produce His_SntC_H132A_ protein; lacZ, *Kana*^*r*^	This study
pET30a*_sntD*_D13A_	Expression vector to produce His_SntD_D13A_ protein; lacZ, *Kana*^*r*^	This study
pET30a*_sntD*_H15A_	Expression vector to produce His_SntD_H15A_ protein; lacZ, *Kana*^*r*^	This study
pET30a*_sntD*_G80A_	Expression vector to produce His_SntD_G80A_ protein; lacZ, *Kana*^*r*^	This study
pET30a*_sntD*_N81A_	Expression vector to produce His_SntD_N81A_ protein; lacZ, *Kana*^*r*^	This study
pET30a*_sntD*_H82A_	Expression vector to produce His_SntD_H82A_ protein; lacZ, *Kana*^*r*^	This study
pET30a*_sntD*_E83A_	Expression vector to produce His_SntD_E83A_ protein; lacZ, *Kana*^*r*^	This study
pET30a*_sntD*_F84A_	Expression vector to produce His_SntD_F84A_ protein; lacZ, *Kana*^*r*^	This study
pET30a*_sntD*_R336A_	Expression vector to produce His_SntD_R336A_ protein; lacZ, *Kana*^*r*^	This study
pET30a*_sntD*_F355A_	Expression vector to produce His_SntD_F355A_ protein; lacZ, *Kana*^*r*^	This study
pET30a*_sntD*_F411A_	Expression vector to produce His_SntD_F411A_ protein; lacZ, *Kana*^*r*^	This study
pET30a*_sntA*_AYY_	Expression vector to produce His_SntA_A510R Y530F Y633F_ protein; lacZ, *Kana*^*r*^	This study
pSET2*_sntA*	pSET2 containing the intact *sntA* gene and its upstream promoter; *Spc*^*r*^	This study
pSET2*_sntB*	pSET2 containing the intact *sntB* gene and its upstream promoter; *Spc*^*r*^	This study
pSET2*_sntC*	pSET2 containing the intact *sntC* gene and its upstream promoter; *Spc*^*r*^	This study
pSET2*_sntD*	pSET2 containing the intact *sntD* gene and its upstream promoter; *Spc*^*r*^	This study

### Bioinformatics analysis

Multiple alignment analysis and phylogenetic analysis of 5’-nucleotidase was performed using CLC Sequence View version 8.0. The amino acid sequence identity analysis was performed by BLAST (available at: https://blast.ncbi.nlm.nih.gov/Blast.cgi). The structural modelling of *S. suis* 5’-nucleotidase was obtained from UniProt (available at: https://www.uniprot.org/) generated by AlphaFold2. The structural alignment analysis between mammal CD73 structure (PDB ID: 4H1S.1.A) with SntA (AlphaFold PDB ID: A0A2K1SX24), SntB (AlphaFold PDB ID: D5AHM7.1.A), SntC (AlphaFold PDB ID: A0A2K1T1H0), and SntD (AlphaFold PDB ID: D5AFY5) was analysed by PyMOL version 1.2r3pre and SWISS-MODEL (available at: https://swissmodel.expasy.org/). The conserved domains of all the proteins from several species were searched using CD-Search (available at: https://www.ncbi.nlm.nih.gov/Structure/cdd/wrpsb.cgi), and the output diagrams illustrating the structural features were performed by IBS version 1.0 (available at: http://ibs.biocuckoo.org/online.php).

### Expression and purification of recombinant proteins and mutants

To express recombinant proteins, the *sntA* (Locus_tag: B9H01_RS10070), *sntB* (Locus_tag: B9H01_RS04925), *sntC* (Locus_tag: B9H01_RS07430), and *sntD* (Locus_tag: B9H01_RS10185) genes were amplified from the genomic DNA of *S. suis* SC-19 and digested with restriction enzymes *Bam*H I/*Sal* I, *Bam*H I/*Xho* I, *Bam*H I/*Xho* I, and *Kpn* I/*Xho* I, respectively. The resulting fragments were cloned into a pET30a plasmid. The site-mutagenesis *sntA*, *sntB*, *sntC,* and *sntD* genes were amplified by overlapping PCR and introduced site-mutated with primers, and cloned into pET30a as mentioned above.

Recombinant plasmids were transformed into *E. coli* Rosetta (DE3) competent cells (Tsingke, China). The resulting *E. coli* cells in the mid-log phase were induced by 1 mmol/L isopropyl-β-d-thiogalactoside (IPTG) (Sangon, China) at 16°C for 16 h to express recombinant proteins. His-tagged proteins were purified by Ni-nitrilotriacetic acid (Ni-NTA) resin (Sigma, USA) affinity chromatography. All the proteins were purified by applying gravity-assisted flow to disposable columns. Purified proteins were quantified using a BCA protein assay kit (Beyotime, Shanghai, China) and subjected to 10% SDS-PAGE.

### Construction of gene deletion and site mutant strains

Four markerless single-gene deletion strains retained 6 amino acids at the N-terminal and C-terminal of each gene, and 1 four-gene deletion strain was constructed *via* a two-step procedure as described previously [23]. Take *sntA* single-gene deletion strain, for example, in the first step, primers *sntA*-P1/P2, *sntA*-P5/P6, and *sntA*-P3/P4 were used to amplify the upstream and downstream arms of *sntA*, SCIY cassette, respectively. These three resulting DNA fragments were fused into one fragment using overlapping PCR and transformed into *S. suis* SC-19 by natural transformation to obtain the intermediate strain. In the second step, primers *sntA-1*-P1/P7, and *sntA*-P8/P6 were used to amplify the upstream and downstream arms of *sntA*, respectively. These two resulting fragments were fused and transformed into the intermediate strain by natural transformation to obtain markerless *sntA* gene deletion strain Δ*sntA*. The *sntB*, *sntC* and *sntD* single-gene deletion strains Δ*sntB*, Δ*sntC,* and Δ*sntD* were constructed as described above. The four-gene deletion strain was constructed based on Δ*sntA* through deletion of *sntB*, *sntC* and *sntD* genes one by one.

Four single-site mutant strain *sntA*_*H209A*_, *sntB*_*H161A*_, *sntC*_*H132A*_, *sntD*_*H82A*_ were constructed also as described above with some modifications. Taken *sntA*_*H209A*_ as example, the His-209 residue was transformed to Ala (cac→gca) using primers *sntA-*H209A*-F*/*sntA-*H209A*-R* in order to generate site-mutagenesis *sntA* gene. The N-terminal and C-terminal segments of site-mutagenesis *sntA* gene were amplified by primers *sntA*-P1/*sntA-*H209A*-R* and *sntA-*H209A*-F*/*sntA*-P6, respectively. Subsequently, these two resulting segments were fused into one intact site-mutagenesis *sntA* gene using overlapping PCR and transformed into the intermediate strain mentioned before by natural transformation to obtain site mutant strain *sntA*_*H209A*_. The single-site mutant strains *sntB*_*H161A*_, *sntC_H132A_,* and *sntD*_*H82A*_ were constructed through mutation of the corresponding His residue to Ala (cat→gca) as described above. The four-site mutant strain was constructed based on *sntA*_*H209A*_ through mutation of other three His residues in *sntB*, *sntC,* and *sntD* one by one.

The *sntA*, *sntB*, *sntC,* and *sntD* complementary strains CΔ*sntA*, CΔ*sntB*, CΔ*sntC,* and CΔ*sntD* were constructed as the method mentioned before [24]. To construct *sntA* complementary strain, a DNA fragment containing the entire *sntA* coding sequence and its promoter was amplified by using primers pSET2-*sntA*-F/R. The amplicon was subsequently cloned into *E. coli–S. suis* shuttle vector pSET2, resulting in the recombinant plasmid pSET2:*sntA*. This plasmid was transformed into the makerless *ΔsntA* strain, and the resulting complementary strain C*ΔsntA* was screened on TSA agar plates supplemented with 100 μg/ml spectinomycin (Spc). The other complementary strains CΔ*sntB*, CΔ*sntC,* and CΔ*sntD* were constructed as described above. All the gene deletion strains and complementary strains were verified using genomic PCR and RT-PCR.

### Enzymatic activity assays

Phosphate (Pi) was detected using malachite green reagent following the manufacturer’s recommendations (Biomol Green, Enzo Life Sciences, USA). The reaction was carried out at 37°C in TM buffer (50 mM Tris-HCl, 5 mM MnCl_2_, pH7.5) with increasing concentrations of 0–100 μM ATP (Sigma), ADP (Sigma), and AMP (Sigma) and 0.1 μM recombinant SntA, SntB, SntC, SntD, and their related site-mutagenesis enzymes in a total volume of 50 μL for 30 min. After stopping the reaction with 1 ml of Biomol Green reagent, the samples were incubated at room temperature for 20–30 min to allow the development of the green colour. The Pi concentrations were determined by spectrophotometric absorbance measurements at 620 nm using a standard Pi curve. To investigate the effect of pH, temperature, and cofactors on the reaction, the reaction was carried out at different temperature of 22–52°C in 50 mM Tris-HCl adjusted to different pH values (between 6 and 8.5) containing 50 µM AMP, 5 mM different cofactors, and 0.1 μM recombinant SntA, SntB, SntC, or SntD enzymes. The enzyme kinetics were analysed by incubation of increasing amounts of 0–500 µM AMP for Snts (exception for 0–2, 000 µM AMP for SntD) with a fixed concentration of 0.05 μM Snts enzyme at 37°C in a total volume of 50 μL. The reactions were stopped and detected as described above. Michaelis–Menten curve fitting using nonlinear regression was performed using GraphPad Prism 9 software.

The generation of Ado was determined by HPLC using a Prominence UFLC LC20AD (Shimadzu, Japan) with a reverse-phase InertSustain C18 column (Shimadzu, 4.6 mm × 250 mm, 5 µm). Reactions were performed in TM buffer containing 500 µM AMP and 10 µM recombinant Snts in a total volume of 400 µL at 37°C for 30 min. The enzymatic reaction was stopped by the addition of EDTA to a final concentration of 50 mM. The reaction samples (20 µL) were loaded and eluted in a linear solvent gradient with 100% methanol running from 2% to 70% (2%, 2–50%, 50–65%, 65–70%, 70%, 70–2%, and 2% methanol) in 20 mM NH_4_H_2_PO_4_/30 mM K_2_HPO_4_ buffer at a flow rate of 1 mL/min. AMP and Ado were detected at an absorbance wavelength of 254 nm. Commercial chromatographic grade AMP and Adenosine (Sigma) were used as standards.

### Bacterial enzymatic activity assays

The *S. suis* strains were grown to the middle-log phase and 50 μL *S. suis* strains cultures (2 × 10^9^ CFU/mL) were collected at 2, 800 g for 5 min. The culture precipitate was resuspended in the TM buffer and mixed with 1 mM AMP. A total volume of 400 μL of each compounds was added to the reaction at 37°C for 30 min. Reactions were stopped and detected using both the malachite green reagent and HPLC.

### Whole blood killing assay

Blood-killing assays were performed as described previously [24]. Mixtures of 1 × 10^4^ CFU *S. suis* strain cultures and 450 μL fresh mouse blood were incubated at 37°C for 0.5 h. Exogenous adenosine (10 μM; Sigma), 5’-nucleotidase inhibitor (5’-(α, β-methylene) diphosphate) (500 µM; Sigma), and sterile ddH_2_O as a control were added to these mixtures. Live bacteria were counted by plating serially diluted samples on BHI agar. The survival index of live bacteria was subsequently calculated as CFU _after incubation_/CFU _in original inoculum_. Data are presented as the mean ± standard deviation (SD) from three separate replicates. The experiments were repeated three times.

### Opsonophagocytosis assay

Opsonophagocytosis assays were performed as previously described [24]. PMNs were isolated from heparinized venous blood by using a mouse peripheral blood PMNs isolation kit (Haoyang, China). PMNs were mixed with bacteria at an MOI of 1:10 in RPMI-1640 (Hyclone, USA) supplemented with 20% fresh non-immune mouse serum and incubated at 37°C under 5% CO_2_ for 30 min. Exogenous adenosine (10 μM; Sigma), 5’-nucleotidase inhibitor (5’-(α, β-methylene) diphosphate) (500 µM; Sigma), A2aR antagonist (ZM241385, 1 μM; Sigma), and sterile ddH_2_O (control) were added to the mixtures. The percentage of live bacteria was calculated as (CFU _PMN+_/CFU _PMN-_) × 100%. Data are presented as the mean ± standard deviation (SD) from three separate replicates. The experiments were repeated three times.

### Mouse infection assay

To detect the effect of Ado synthesis on the virulence of *S. suis*, a mouse model was used as described previously [24]. Sixty female 5-week-old specific-pathogen-free (SPF) ICR mice were acquired from the Laboratory Animal Centre of Hangzhou Medical College, Hangzhou, Zhejiang Province, China. These mice were randomly divided into six groups (10 mice per group) and intraperitoneally infected with 3 × 10^8^ CFU/mouse of *S. suis*. The survival rate of mice was recorded at 12 h intervals for 7 days. To evaluate the effect of Ado synthesis on survival in the bloodstream and colonization of distant organs in mice, a total of 72 female 5-week-old SPF ICR mice (six mice per group) were intraperitoneally infected with 1 × 10^8^ CFU/mouse of *S. suis*. Bacterial counts in the blood, brain, kidneys, and lungs were collected at 12 and 24 h post infection (hpi). Bacterial colonies in various tissues were analysed on BHI agar plates, as described previously. Flowcharts of animal study were shown in Figs. S1 and S2. All the mice in each group were housed in separate cages (4–5 mice per cage) at 21–26°C and 50–60% humidity.

## Results

### Bioinformatics analysis

**Figure 1.**
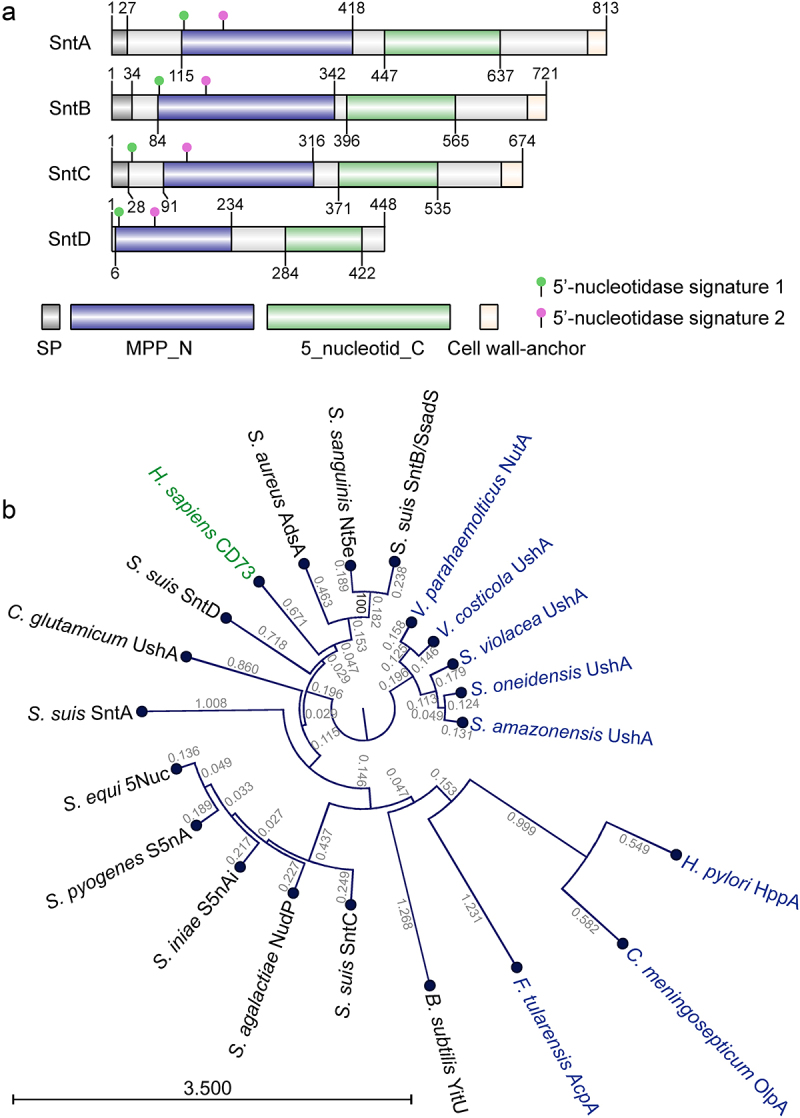
Structural and phylogenetic analysis of *S. suis* 5’-nucleotidases SntA, SntB, SntC, and SntD.

5’-Nucleotidase is a ubiquitous enzyme found in a wide variety of species and is characterized by two distinct motifs: [LIVM]-x-[LIVM](2)-[HEA]-[TI]-x-D-x-H-[GSA]-x-[LIVMF] and [FYPH]-x(4)-[LIVM]-G-N-H-E-F-[DN] [5]. In the *S. suis* SC19 genome, four putative 5’-nucleotidases, SntA, SntB, SntC, and SntD, were identified based on these motifs. All the putative Snts had two conserved domains: N-terminal MPP_superfamily metallophosphatase domain (MPP_N) and C-terminal 5’-nucleotidase domain (5_nucleotid_C), and three harboured a C-terminal LPXTG sorting motif, with the exception of SntD (Figure 1a), suggesting that their mature products are localized in the cell wall envelope [12]. Phylogenetic analysis juxtaposed these *S. suis* putative nucleotidases with their counterparts from mammals, and both Gram-positive and Gram-negative bacteria elucidated evolutionary relationships (Figure 1b). Branch lengths (b) from the phylogenetic tree and amino acid sequence identity (id) using BLAST revealed a substantial identity between SntB with known 5’-nucleotidases *S. suis* Ssads (b: 0; id: 100%), *S. sanguinis* Nt5e (b: 0.427; id: 62.69%), and SntC with *S. equi* 5Nuc (b: 0.494; id: 59.56%), *S. pyogenes* S5nA (b: 0.498; id: 58.93%), *S. agalactiae* NudP (b: 0.476; id: 57.86%), *S. iniae* S5nAi (b: 0.493; id: 57.58%). However, SntA and SntB’s identity to each other and to *S. violacea* UshA was relatively low, underlining distant phylogenetic connections despite over 30% sequence similarity (Figure 1b, Table 2). In contrast, internal comparisons among SntA, SntB, SntC, and SntD revealed minimal resemblance to each other and to known 5’-nucleotidases, with sequence identities below 30% and/or branch lengths above 1 (Figure 1b, Table 2). These data underscore the composition of *S. suis* SC-19 5’-nucleotidases, which, despite their low sequence identity, share analogous structural domains.

(a) Diagrams illustrating the structural features of SntA, SntB, SntC, and SntD, illustrating shared 5’-nucleotidases analogous domains: N-terminal MPP_superfamily metallophosphatase domain (MPP_N), C-terminal 5’-nucleotidase domain (5_nucleotid_C), and three of them harboured C-terminal LPXTG sorting motif except for SntD. Four *S. suis* 5’-nucleotidases harboured two signatures, signature 1: [livm]-x-[LIVM](2)-[hea]-[ti]-x-D-x-H-[gsa]-x-[livmf] (green solid point), and signature 2: [fyph]-x(4)-[livm]-G-N-H-E-F-[dn] (purple pink solid point). (b) Phylogenetic tree of SntA, SntB, SntC, and SntD alongside previously identified 5’-nucleotidases from mammals (green), Gram-positive bacteria (black), and Gram-negative bacteria (blue).

**Table 2.** Sequence identity alignment of the amino acid (a.a.) sequences of *S. suis* SC-19 SntA, SntB, SntC, and SntD with known 5’-nucleotidases from other species.

			a.a. identity (%)			
Bacterial specials and 5’-nucleotidases	Genbank ID	a.a. Length	SntA	SntB	SntC	SntD
*S. suis* SntA^a^	WP_012027972.1	813	100	34.3	26.73	22.59
*S. suis* SntB^a^	WP_032498873.1	714	34.3	100	27.61	27.59
*S. suis* SntC^a^	WP_012775266.1	674	26.73	27.61	100	20.8
*S. suis* SntD^a^	WP_012027993.1	448	22.59	27.59	20.8	100
*S. aureus* AdsA^a^	WP_061821283.1	772	26.27	41.49	22.79	28.9
*S. sanguinis* Nt5e^a^	AFK32764.1	718	29.52	62.69	26	28.54
*S. agalactiae* NudP^a^	CDN66659.1	690	25.24	23.81	57.86	23.06
*S.equi* 5Nuc^a^	AEJ25391.1	668	25.83	26.57	59.56	20.26
*S. pyogenes* S5nA^a^	Q9A0A2	670	22.34	23.18	58.93	20.94
*S. iniae* S5nAi^a^	WP_003099850.1	676	24.24	25.65	57.58	19.69
*C. glutamicum* UshA^a^	WP_011896359.1	651	21.84	24.7	29.2	23.23
*B. subtilis* Yitu^a^	P70947.1	270	0	0	0	0
*V. parahaemolyticus* NutA^b^	P22848.2	560	23.36	27.64	25	25.72
*V. costicola* UshA^b^	WP_102505627.1	557	24.34	28.62	23.63	26.35
*S. violacea* UshA^b^	WP_041419915.1	569	23.91	30.09	23.65	26.49
*S. amazonensis* UshA^b^	WP_011760134.1	571	23.73	29.1	21.24	25.69
*S.oneidensis* UshA^b^	Q8EFH1	569	26.03	28.79	22.59	25.69
*H. pylori* HppA^b^	Q6UC93	245	0	0	0	0
*C. meningosepticum* OlpA^b^	O08351	267	0	29.58	0	0
*F. tularensis* AcpA^b^	WP_003027314.1	514	0	0	0	0
*H. sapiens* CD73^c^	AAH65937.1	574	21.27	26.56	24.12	27.38

a: Gram-positive bacteria; b: Gram-negative bacteria; c: Eukaryotes.

### Identification of adenosine synthase

**Figure 2.**
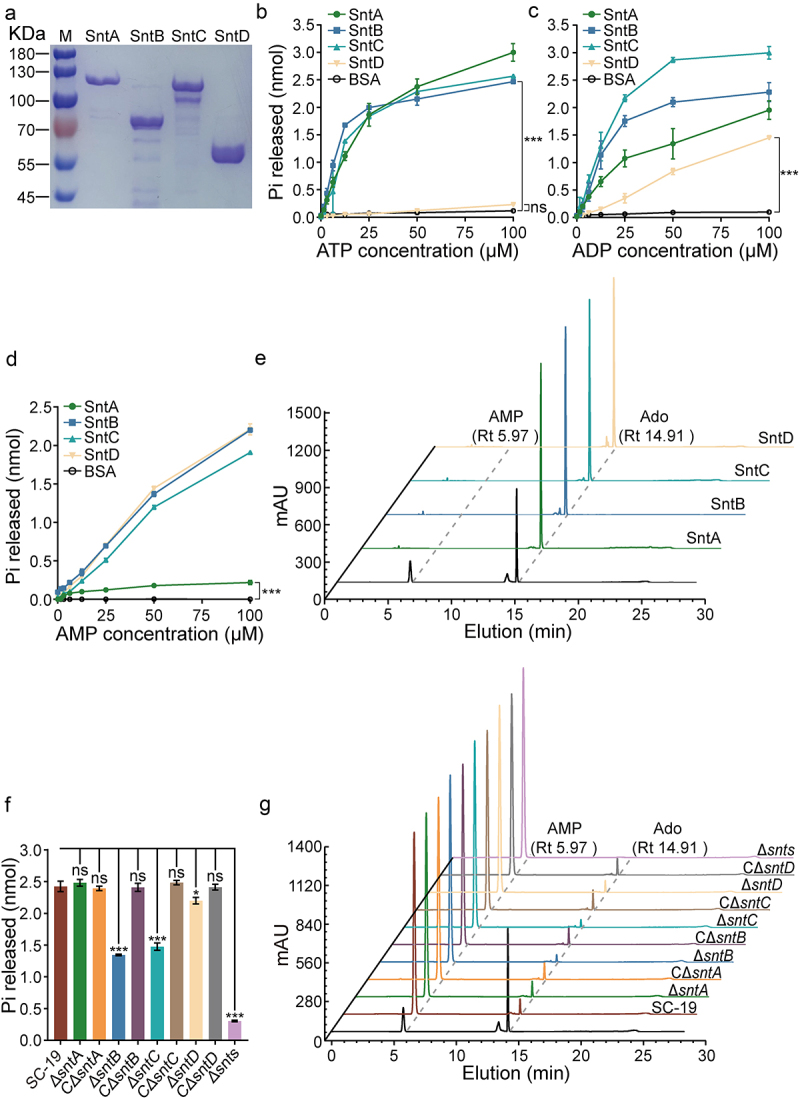
Enzymatic activity assay of *S. suis* 5’-nucleotidases.

The N-terminal His-tagged SntA (93 KDa), SntB (79 KDa), SntC (76 KDa), and SntD (55 KDa) were characterized following their expression in *E. coli*, purified *via* affinity chromatography, and confirmed by SDS-PAGE analysis (Figure 2a). Enzymatic assays using the malachite green reagent method revealed the differential abilities of these enzymes to produce inorganic phosphate (Pi) from ATP, ADP, and AMP, with notable increases in Pi production from ATP by SntA, SntB, and SntC, an unremarkable increase in SntD (Figure 2b), and significant increases in ADP and AMP across all enzymes, indicating their varying capacities to convert these substrates into adenosine (Figure 2c,d). Further analysis using HPLC confirmed these findings, particularly for AMP hydrolysis (Figure 2e). Gene deletion strains for each nucleotidase (Δ*sntA*, Δ*sntB*, Δ*sntC,* and Δ*sntD*), a collective deletion strain (Δ*snts*), and complementary strains for each deletion (CΔ*sntA*, CΔ*sntB*, CΔ*sntC,* and CΔ*sntD*) were constructed and verified using genome-PCR and RT-PCR (Fig. S3). The AMP hydrolysis ability of natural SntA, SntB, SntC, and SntD in *S. suis* SC-19 was analysed using both malachite green reagent and HPLC. The results showed that the Ado generation capacity was significantly reduced in Δ*sntB*, Δ*sntC*, Δ*sntD*, and strongly decreased in Δ*snts*, with no significant change observed in the Δ*sntA* strain (Figure 2f–g). This suggests an additive role of SntB, SntC, and SntD in adenosine synthesis, which is supported by the lack of change in adenosine production among *S. suis* SC-19 and all complementary strains, indicating that naturally occurring SntB, SntC, and SntD retain their functional capacity for AMP hydrolysis. Consequently, it was demonstrated that four recombinant nucleotidases, along with their natural counterparts excluding SntA, can hydrolyse ATP, ADP, and AMP to various extents, thereby contributing to the production of the immunosuppressive molecule, adenosine.

(a) The purification of recombinant 5’-nucleotidases SntA (residues 28–813), SntB (residues 35–714), SntC (residues 29–674), and SntD (residues 1–448) visualized on an SDS-PAGE gel. b–e. Evaluation of the hydrolysis activities of recombinant *S. suis* 5’-nucleotidases towards ATP (b), ADP (c), and AMP (d and e) using malachite green reagent (b–d) and HPLC (e). f–g. Evaluation of AMP hydrolysis by natural *S. suis* 5’-nucleotidases using malachite green reagent (f) and HPLC (g). The data were represented as means ± standard deviations (SD) from three independent experiments. Statistical significance was determined by two-way ANOVA with Dunnett’s multiple comparison test (a–d) and unpaired t-test (f), denoted by asterisks (***p<0.001; ns, *p*>0.05).

### The pH, temperature, metal cofactors, and kinetics specificity of Snts

**Figure 3.**
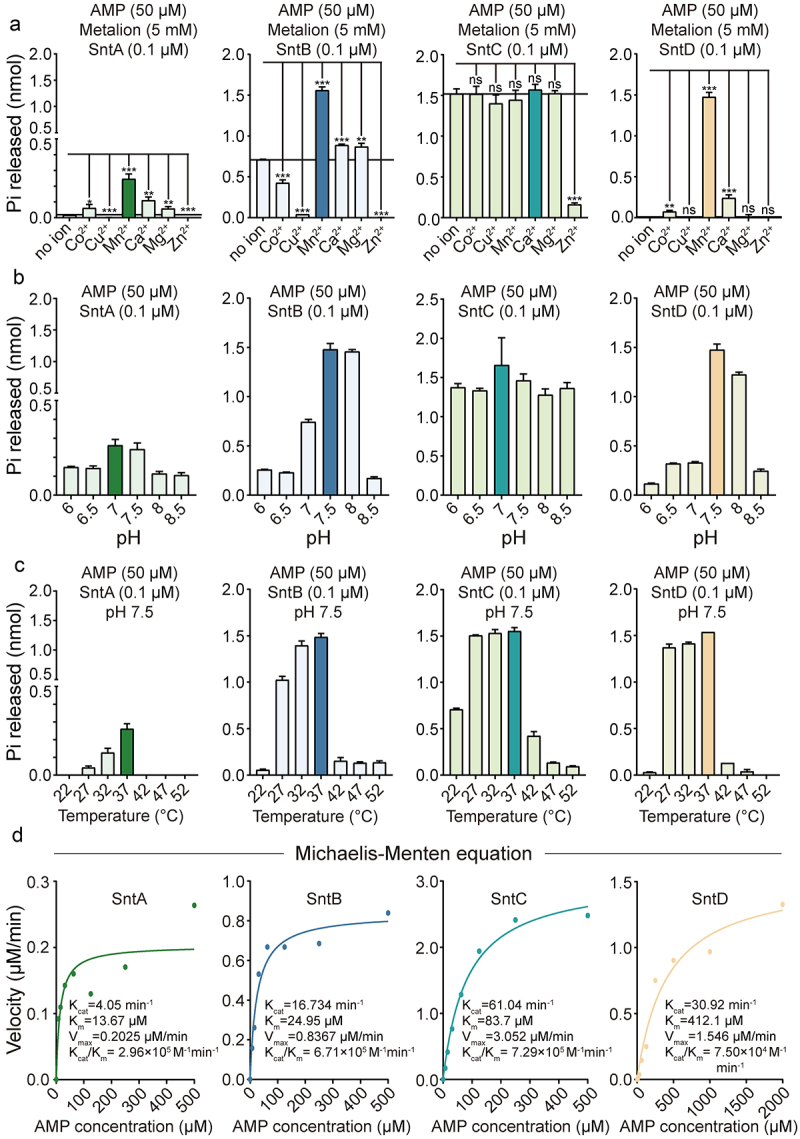
Determining the optimal conditions for ***S. suis* 5’-nucleotidases**.

The enzymatic activity of recombinant Snt proteins was evaluated under various conditions, revealing distinct preferences for metal cofactors. Specifically, SntA activity was enhanced by Co^2+^, Mn^2+^, Ca^2+^, and Mg^2+^, whereas SntB was stimulated by Mn^2+^, Ca^2+^, Mg^2+^, and SntD by Co^2+^, Mn^2+^, Ca^2+^, and SntC showed no dependency on cations, indicating that Mn^2+^ is the necessary metal cation for the enzymatic activity across Snts (Figure 3a). In contrast, all the Snts displayed minimal to no activity in the presence of 5 mM Zn^2+^. The enzymes demonstrated optimal pH values for activity at pH 7.0 for SntA and SntC, and pH 7.5 for SntB and SntD, aligning closely with the pH of blood and extracellular fluids of the body (Figure 3b). The optimal temperature for enzymatic activity was 37°C, which approximates the human body temperature and is slightly below that of pigs (Figure 3c). Kinetic analyses following the Michaelis–Menten model highlighted differences in the initial rate of Pi release with varying concentrations of AMP. The kinetic parameters were determined as *Km* and *Vmax* for each enzyme: SntA exhibited a *K*_*m*_ of 13.67 μM and *Vmax* of 0.2025 μM/min, SntB a *K*_*m*_ of 24.95 μM and *Vmax* of 0.8367 μM/min, SntC a *K*_*m*_ of 83.7 μM and *Vmax* of 3.052 μM/min, and SntD a *K*_*m*_ of 412.1 μM and *Vmax* of 1.546 μM/min. The *K*_*cat*_/*K*_*m*_ of SntA, SntB, SntC, and SntD were 2.96 × 10^5^ M^−1^min^−1^, 6.71 × 10^5^ M^−1^min^−1^, 7.29 × 10^5^ M^−1^ min^−1^ and 7.50 × 10^4^ M^−1^min^−1^, respectively (Figure 3d). These findings underscore the nuanced catalytic efficiency of Snts towards AMP, with SntC being the most efficient, followed by SntB, SntA, and SntD.

(a) The effect of different metal ions Co^2+^, Cu^2+^, Zn^2+^, Mn^2+^, Ca^2+^, and Mg^2+^ on the enzymatic activity. (b) The effect of varying pH levels (ranging from 6 to 8.5) on the enzymatic activity. (c) The effect of temperature changes (22–52 °C) on the enzymatic activity. (d) The Michaelis–Menten kinetics of the enzymes, analysed with increasing AMP concentrations were plotted and fitted using nonlinear regression in GraphPad prism 9 software. The data were represented as means ± standard deviations (SD) from three independent experiments. Statistical significance was determined by unpaired t- test and denoted by asterisks (***p < 0.001; **0.001).

### Ado generation activity analysis of recombinant and natural Snts mutations

**Figure 4.**
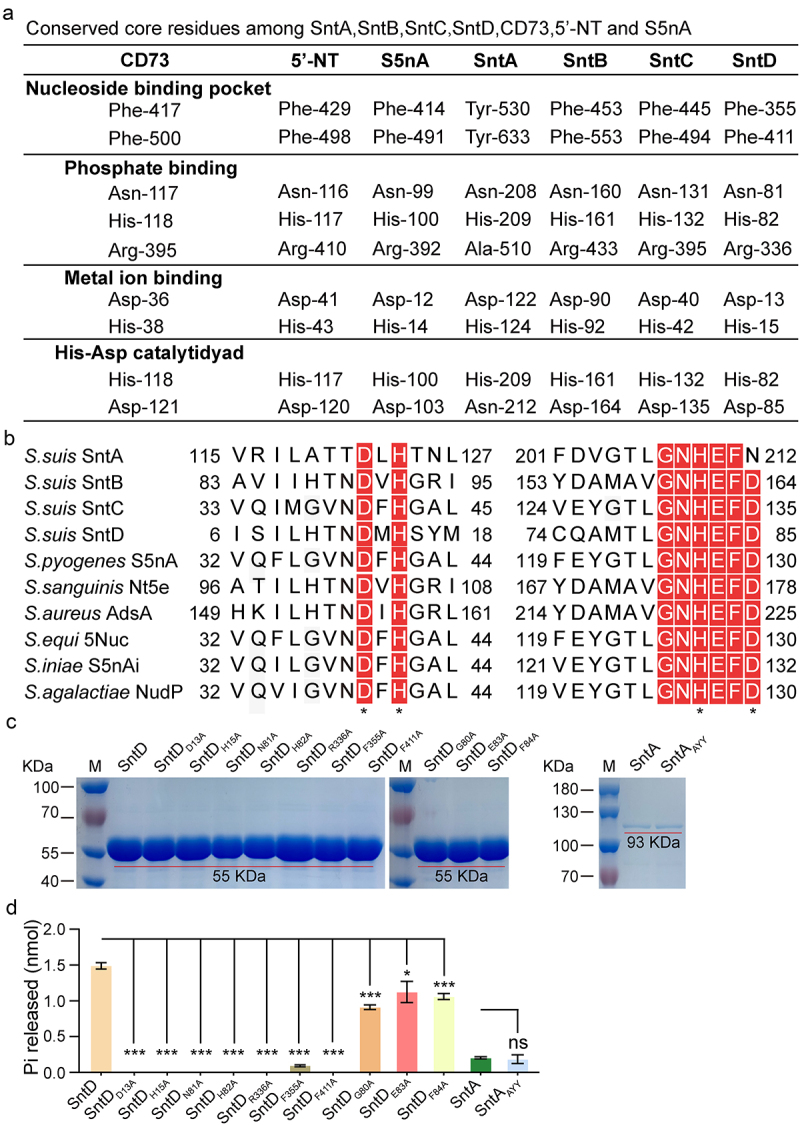
Analysis of catalytic sites in ***S. suis*** **5’-nucleotidases**.

**Figure 5.**
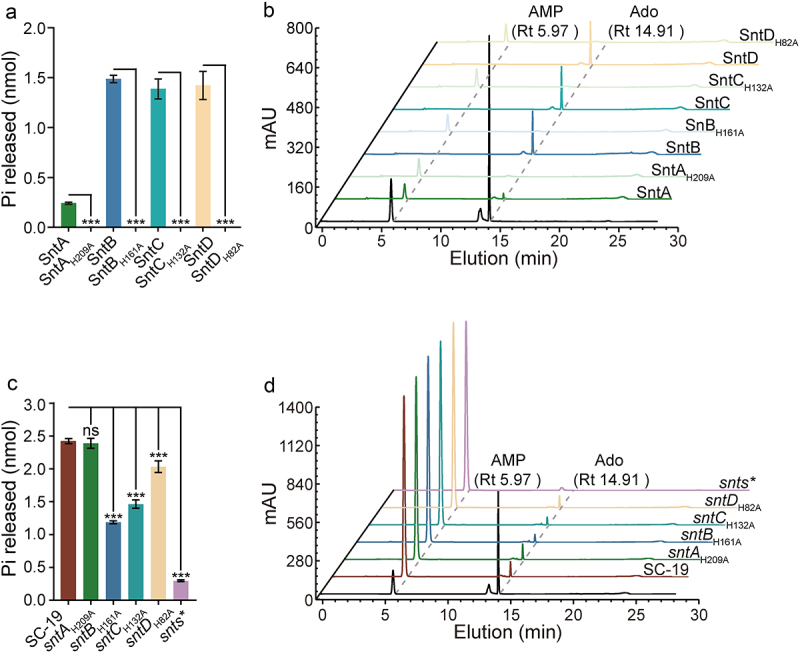
Assessing the enzymatic activity of mutant *S. suis* 5’-nucleotidases. a–b. Analysis of AMP hydrolysis by recombinant mutant proteins SntAH209A, SntBH161A, SntCH132A, and SntDH82A using the malachite green reagent (a) and HPLC (b). c–d. Analysis of AMP hydrolysis by *S. suis* strains subjected to site-directed mutagenesis (*sntAH209A*, *sntBH161A*, *sntCH132A,* and *sntDH82A*) using the malachite green reagent (c) and HPLC (d). The data were represented as means ± SD from three independent experiments. Statistical significance was determined by two-way ANOVA of Dunnett’s multiple comparisons test, denoted by asterisks (***p < 0.001; *0.01 < 0.05; ns,=“‘ *p*=’‘ contenteditable=’true”>0.05).< 0.05;>).

Multiple sequence alignments of SntA, SntB, SntC, SntD, CD73 [25], S5nA [26], and *E. coli* 5’-nucleotidase [27] revealed conserved features among these enzymes. Notably, the substrate-binding pocket, featuring phenylalanine residues in SntB, SntC, and SntD, contrasts with the tyrosine residues in SntA. The His–Asp catalytic dyad, which is critical for enzymatic function, is conserved across SntB, SntC, and SntD, with a notable deviation in SntA, where Asn replaces Asp. Metal ion- and phosphate-binding sites were preserved across all Snts, indicating a shared structural framework for their enzymatic activity (Figure 4a). Additionally, other essential residues from the 5’-nucleotidase signatures, GNHEFD, were conserved across these enzymes (Figure 4b). Structural alignment analysis between *S. suis* 5’-nucleotidase with the mammal CD73 revealed different coverage rates for Snts (SntA: 62%; SntB: 63.9%; SntC: 71.5%; SntD: 98%), with SntD exhibiting the highest coverage. Mutation analysis of SntD, focusing on its catalytic, metal ion binding, phosphate binding, and nucleotide-binding sites, confirmed the critical roles of these residues in the generation of Ado (Figure 4c–d). Moreover, Gly-80, Glu-83, and Phe-84 in the 5’-nucleotidase signatures were first discovered to be important for AMP hydrolysis, but not for the key cores (Figure 4d). Interestingly, mutation analysis of SntA with altered residues did not account for its limited 5’-nucleotidase activity, suggesting that other factors are at play (Figure 4d). These results suggest that the enzymatic mechanism is similar to that of SntB, SntC, SntD, and the known 5’-nucleotidases. Further experiments with mutant recombinant proteins in which histidine residues critical for activity were altered to alanine (SntA_H209A_, SntB_H161A_, SntC_H132A_, and SntD_H82A_) showed a complete loss of Ado generation, as confirmed by both the malachite green assay and HPLC (Figure 5a–b). Similar results were observed in single site-mutagenesis strains (*sntA*_*H209A*_, *sntB*_*H161A*_, *sntC*_*H132A*_, *sntD*_*H82A*_), confirming the essential nature of these histidine residues for the enzymatic activity of Snts (Figure 5c–d). Collectively, these data highlight the crucial role of specific residues in the 5’-nucleotidase activity of Snts and their similarity to known 5’-nucleotidases, highlighting the conservation of enzymatic mechanisms across different species.

(a) Conserved core residues among SntA, SntB, SntC, and SntD were predicted by multiple sequence alignment based on CD73, *E. coli* 5’-nucleotidase, and S5nA. (b) Multiple sequence alignment of two signature sequences critical for 5’-nucleotidase activity across Snts and other established 5’-nucleotidases. The sites of his-asp catalytic dyad and metal ion binding are marked above using asterisks (*). (c) Purification of recombinant mutant SntD and SntA proteins, verified by SDS-PAGE gel analysis. (d) Evaluation of the hydrolysis activity of the mutant proteins SntD and SntA towards AMP. The data were represented as means ± SD from three independent experiments. Statistical significance was determined by unpaired t-test, denoted by asterisks (***p < 0.001; *0.01).

### Ado generation increases the anti-opsonophagocytosis of S. suis

**Figure 6.**
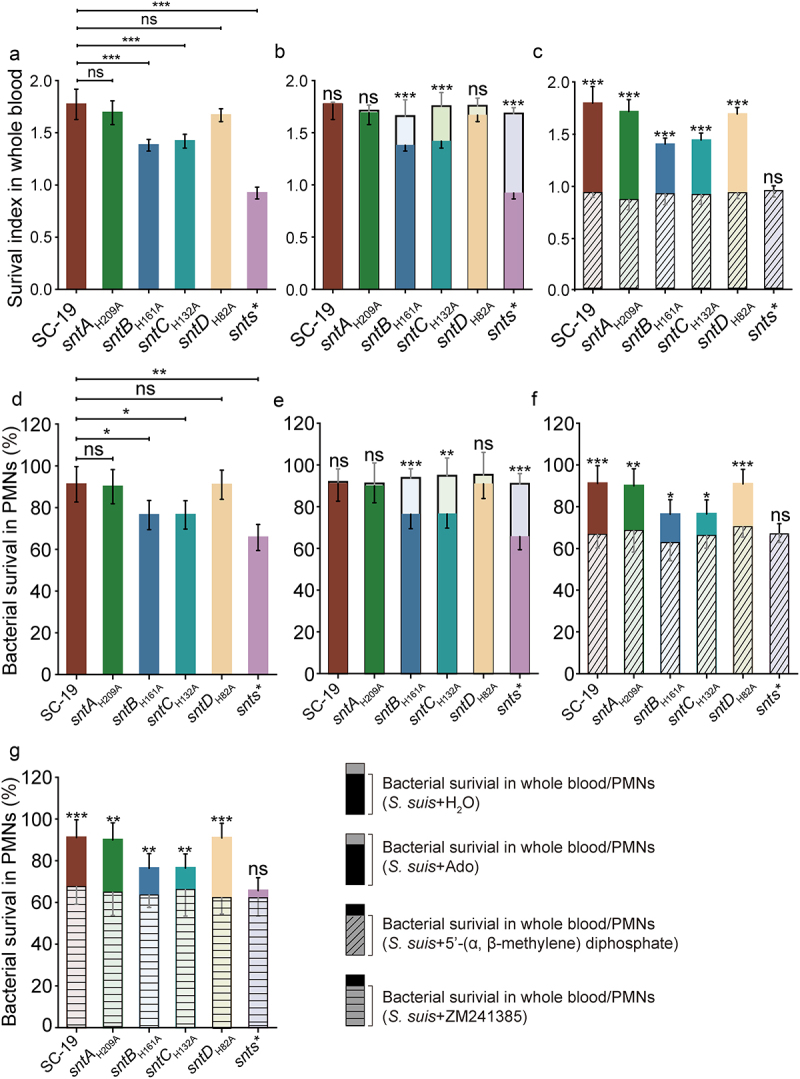
Impact of adenosine and inhibitors on *S. suis* survival in blood and PMNs.

To assess the impact of Ado generation on the anti-opsonophagocytosis capabilities of *S. suis*, whole blood and PMNs killing assays were performed. In a mouse whole-blood killing assay, the survival rates of *sntB*_*H161A*_, *sntC*_*H132A*_ and particularly the snts* mutant strains, were significantly lower than those of wild-type *S. suis* SC-19. No obvious differences in survival were observed between the *sntA*_*H209A*_ and *sntD*_*H82A*_ mutants and wild-type strain (Figure 6a). The addition of exogenous adenosine to *S. suis* strains resulted in increased survival across all the site-mutagenesis strains, with the *sntB*_*H161A*_, *sntC*_*H132A*_ and snts* strains showing significant increases of 16.75%, 19.03%, and 45.18%, respectively (Figure 6b). The application of a 5’-nucleotidase inhibitor (5’-(α, β-methylene) diphosphate) led to a marked reduction in the survival of SC-19 and the single-mutagenesis strains, whereas the snts* strain showed no significant change (Figure 6c). Similar results were observed in the PMNs killing assay, regardless of the addition of exogenous adenosine or 5’-nucleotidase inhibitor (Figure 6d–f). These results demonstrate that Snts collectively contribute to bacterial survival in both whole blood and PMNs, although the roles of SntB and SntC, which exhibit strong enzymatic activity, are more critical than those of SntA and SntD, which exhibit limited activity. Adenosine decreases phagocytic activity in macrophages by suppressing the generation of nitric oxide [28], superoxide [29,30] and pro-inflammatory cytokines [31] through signalling *via* the A2a receptor. The use of an A2aR antagonist to investigate the role of A2aR in anti-opsonophagocytosis resulted in reduced survival rates for all strains, except for the snts* mutant strain (Figure 6g), indicating that the Ado generation activity of Snts enhances bacterial survival in PMNs through the Ado-A2aR pathway, without ruling out the involvement of other Ado receptors. In summary, the capacity of Snts to generate adenosine promotes bacterial survival in whole blood and PMNs by engaging the Ado-A2aR pathway, highlighting a direct correlation between adenosine generation and augmentation of bacterial resistance against opsonophagocytosis.

a–c. Survival of *S. suis* in mouse blood following supplementation with equal volume of sterile ddH_2_O (a), 10 μM adenosine (b), or 500 μM 5’-nucleotidase inhibitor (5’-(α, β-methylene) diphosphate) (c). d–g. Survival of *S. suis* in mouse PMNs following supplementation with equal volume of sterile ddH_2_O (d), 10 μM adenosine (e), 500 μM 5’-nucleotidase inhibitor (5’-(α, β-methylene) diphosphate) (f), or 1μM A2aR antagonist (ZM241385) (g). The data were represented as means ± standard deviations (SD) from three independent experiments. The data were represented as means ± SD from three independent experiments. Statistical significance was determined by unpaired t-test, denoted by asterisks (***p < 0.001; **0.001).

### Ado generation increases the S. suis survival and virulence in mouse

**Figure 7.**
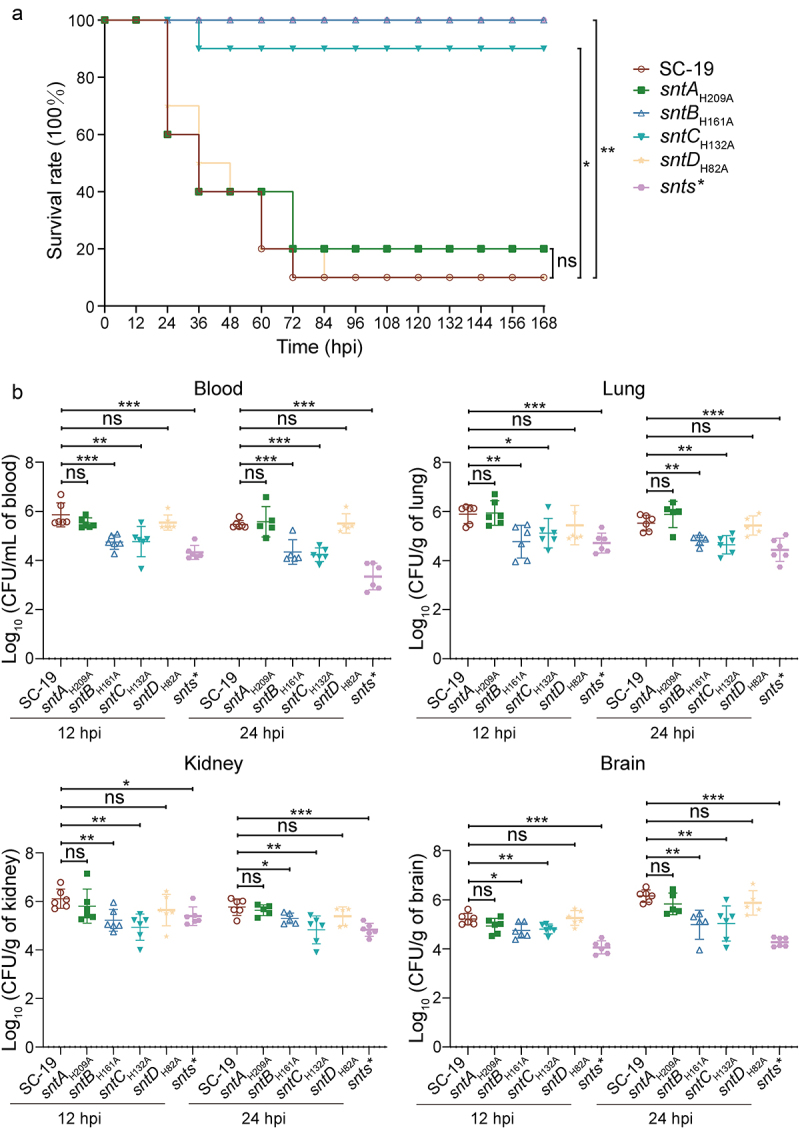
Evaluating the role of 5’-nucleotidases in *S. suis* pathogenicity using mouse infection models.

**Initial observations confirmed that there was no significant** difference in the growth rate between the wild-type and mutant strains ***sntA*_*H209A*_, *sntB*_*H161A*_, *sntC*_*H132A*_, *sntD*_*H82A*_** (Fig. S4). The direct influence of the Ado generation on *S. suis* virulence was further **examined** using mouse infection models. Groups of 10 healthy SPF mice were intraperitoneally infected with 3 × 10^8^ CFU of various *S. suis* strains. The outcomes revealed significant differences in mortality rates at 72 hpi, with nine mice infected with SC-19 and ***sntD*_*H82A*_**, and eight mice infected with ***sntA*_*H209A*_** succumbing to the infection. In contrast, no or minimal mortality was observed with ***sntB*_*H161A*_**, ***sntC*_*H132A*_**, and snts* within 168 hpi, indicating survival rates of 100%, 90%, and 100% for these strains, respectively, compared with 10% and 20% for SC-19 and ***sntA*_*H209A*_** (Figure 7a). These findings revealed that mutations affecting Ado generation in ***sntB*_*H161A*_**, ***sntC_H132A_,*** and snts* significantly reduced mortality compared with the parental SC-19 strain, suggesting a critical role for Ado generation in *S. suis* virulence. *sntA*_*H209A*_ and *sntD*_*H82A*_ also reduced mortality despite of no significant difference comparing with *S. suis* SC-19. Further analysis of intraperitoneal infections of groups of seven mice with 1 × 10^8^ CFU of *S. suis* strains, followed by assessments of blood and organ colonization at 12 and 24 hpi. The colonization efficiency of ***sntB*_*H161A*_**, ***sntC*_*H132A*_** and snts* exhibited marked reductions in blood, lung, brain, and kidney colonization compared to that of SC-19, particularly for the snts* strain, which displayed the most pronounced attenuation (Figure 7b). No significant differences were observed between ***sntA*_*H209A*_ and *sntD*_*H82A*_** comparing with *S. suis* SC-19. These findings underscore that the Ado generation capability of SntB and SntC is essential for the virulence, survival in the blood, and organ colonization of *S. suis*. Additionally, these results suggest a direct positive correlation between the Ado generation capacity and the ability of *S. suis* to survive and colonize the host, contributing to the lethality rate of infected mice, which highlights the importance of Ado generation in the pathogenicity and host interaction of *S. suis*.

(a) Survival analysis of 5-week-old female ICR mice following intraperitoneal infection with 3 × 10^8^ CFU/mouse of *S. suis* SC-19 and its site-mutagenesis strains *sntAH209A*, *sntBH161A*, *sntCH132A* and *sntDH82A*. Each group consisted of 10 mice. Significant difference in survival between different groups was analysed by log rank (mantel-cox) test for trend. (b) Quantification of bacterial loads in blood, brain, lung, and kidney. Six mice per group were intraperitoneally infected with 1 × 10^8^ CFU/mouse of *S. suis*. Results were shown as log_10_ of recovered bacterial counts (CFU/mL in blood and CFU/g in organs), excluding abnormal death. Statistical significance was determined by unpaired t-test, denoted by asterisks (***p < 0.001; **0.001).

## Discussion

**Figure 8.**
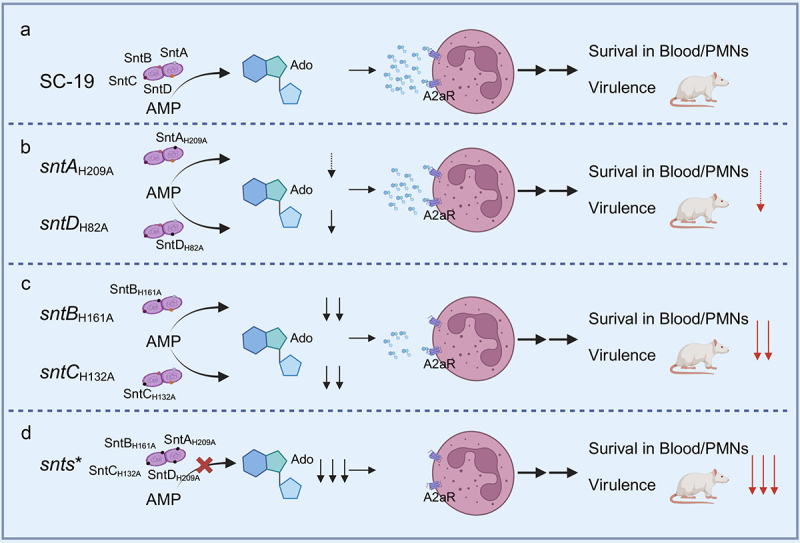
Schematic representation of 5’-nucleotidase enzymatic activity and its contribution to *S. suis* virulence. The figure illustrates how cell wall-anchored 5’-nucleotidases in *S. suis* additively generate the immunosuppressive molecule adenosine (Ado) with varying efficiency, and how this production directly influences the bacterium’s ability to survive in the blood and PMNs, as well as its virulence in mouse models (a–d). SntA and SntD, which have a lower efficiency in generating Ado, have been shown to play a minimal role in affecting the bacterium’s susceptibility to blood and PMNs and its overall virulence (b). Conversely, the pronounced Ado production capabilities of SntB and SntC are critical; their absence significantly diminishes survival in the blood and PMNs, as well as virulence in mouse models (c), underscoring the vital role of effective Ado generation by these enzymes in *S. suis* pathogenicity.

Adenosine (Ado), synthesized by 5’-nucleotidases, plays a dual role in modulating inflammation during acute infections by mitigating excessive immune responses; however, it may also compromise the host’s antimicrobial responses. This delicate equilibrium between ATP/ADP and Ado concentrations is crucial for the maintenance of immune homoeostasis [32]. *S. suis* is an important swine bacterial pathogen that can be transmitted to humans and is the leading cause of severe systemic diseases such as meningitis and septicaemia with sudden death in piglets, and STSLS was first reported in humans in 2005 [21,33]. Despite their significance, the roles and mechanisms of 5’-nucleotidases in the virulence of *S. suis* remain poorly understood. Our study shows that four cell wall-anchored 5’-nucleotidases of *S. suis* effectively catalyse the conversion of ATP and ADP, demonstrating a correlation with increased susceptibility in the blood and PMNs, and enhanced survival and virulence *in vivo* (Figure 8).

5’-Nucleotidases can be found in a wide variety of species, including mammals, protozoa, plants, fungi, and bacteria, and use AMP and ADP, and in some cases ATP, to produce the immunosuppressive Ado, which dampens pro-inflammatory immune responses [34]. Several streptococcal 5’-nucleotidases have been functionally characterized, including *S. sanguinis* Nt5e [13], *S. agalactiae* NudP [35], *S. pyogenes* S5nA [26] from human pathogenic species, and *S. iniae* S5nAi [36], *S. suis* Ssads [37], and *S. equi subsp. zooepidemicus* 5Nuc [15] from animal pathogens that occasionally cause infections in humans [32]. The zoonotic *S. suis* SC-19 harboured four 5’-nucleotidases (Snts), SntA, SntB, SntC, and SntD, which share analogous domains but possess low sequence identity (Figure 1 and Table 2). These enzymes additively process ATP, ADP, and AMP into Ado, with SntB and SntC exhibiting high activity, and SntA and SntD showing limited activity *in vivo* (Figure 2). Multiple sequence alignment of each Snt with CD73 [25], *E. coli* 5’-nucleotidase [27] and S5nA [26] has revealed that the substrate-binding pocket, His–Asp catalytic dyad, metal ion binding, and phosphate-binding sites are fully conserved in recombinant SntB, SntC, SntD, and all the reported 5’-nucleotides [32], suggesting that the enzymatic mechanism is similar for 5’-nucleotidases. In addition, the structural alignment analysis between CD73 with *S. suis* 5’-nucleotidases reveals a higher identity of SntB (34.5%), SntC (34.7%) than SntA (24.7%), SntD (27.63%), suggesting that the different enzymatic activity of *S. suis* 5’-nucleotidases may be attributed to the different structural identity. Besides, at a concentration of 0.1 μM SntA (which nearly releases 0.25 nmol Pi) was found to produce less adenosine than SntD (which nearly releases 1.5 nmol Pi) when both enzymes were exposed to an equal amount of 50 μM AMP *in vitro* (Figures 2d and 3a–c). This outcome is surprising given that SntA’s catalytic efficiency was higher than that of SntD (Figure 3d). The likely explanation for this discrepancy is the significantly lower Vmax of SntA (0.2025 μM/min) compared to SntD (1.546 μM/min). This suggests that the maximum amount of product that can be produced by SntA is limited in comparison to SntD, even under the same conditions. Consequently, the deletion of *sntsD* had a detectable effect whereas the deletion of *sntsA* did not (Figure 2f). It is important to note that the relative expression levels of SntD and SntA in *S. suis* are currently unknown. Evenly, SntA exhibits no detectable activity *in vitro* in previous report [38]. Another previous study report that His-117 (His-209 in SntA) is important in the catalytic activity of *S. enterica* [39] and *E. coli* [40] CpdB which belong to metallophosphatase family, and Tyr-440 and Tyr-544 (Tyr-530, Tyr-633 in SntA) can form a sandwich with the nitrogen base of substrates such as 3’-AMP [39,41]. The amino acid sequence identity between SntA with *S. enterica* CpdB, *E. coli* CpdB are 45.82% and 46.02%, respectively, indicating that SntA is more likely to belong to the CpdB-like metallophosphatase family better than 5’-nucleotidase family.

Despite some differences in substrate specificity, pH range, metal ion requirements, and catalytic efficiency, all characterized 5’-nucleotidases can hydrolyse AMP to immunosuppressive Ado, which dampens pro-inflammatory immune responses and hydrolyzes dAMP to dAdo, triggering caspase-3-dependent apoptosis in macrophages and preventing phagocytic killing of the bacteria [32]. Portions of 5’-nucleotidases, such as NudP [35,37], AdsA [12], S5nAi [42], S5nA [43], and Nt5e [13] have been shown to convert AMP to Ado and relate to survival in host blood and specific tissues and virulence in animal models of infection. However, the effect of 5’-nucleotidases on virulence is attributed to their enzymatic activity or other mechanisms, such as protein–protein interactions. In this study, to emphasize the importance of enzyme activity, site-mutagenesis strains with deletion of AMP hydrolysis capacities to different extents were used to illustrate the direct relationship between Ado generation activity and virulence *in vitro* and *in vivo*. This indicates that 5’-nucleotidases not only contribute to survival in blood, PMNs, and virulence in mouse models directly, but there is also a positive correlation between their Ado generation activities and survival in blood, PMNs, and mice (Figures 6 and 7), clearly laying out the importance of 5’-nucleotidases in virulence *in vitro* and *in vivo*.

In *S. suis* serotype 2, SsnA plays a key role in neutrophil extracellular trap (NET) degradation, resulting in a significant susceptibility against the antimicrobial effect mediated by NETs, although EndAsuis can also degrade NET [44]. Nucleotidases usually work in synergy with nucleases to generate dAdo and have distinct activities [32]. *S. suis* serotype 9 NT (100% identity with SntB) can convert DNA-derived deoxyadenosine monophosphate (dAMP) into 2’-deoxyadenosine (dAdo) to trigger caspase-3-dependent death of mouse macrophages. Further observations have provided direct evidence that *S. suis* NT synthesizes dAdo in mouse blood, which causes monocytopenia in mouse blood *in vivo*. In addition, the *in vivo* transcriptome analysis in mouse blood shows that the inhibitory effect of NT on immune responses and neutrophil functions may be mediated through the generation of Ado [45]. In conclusion, *S. suis* serotype 2 can produce Ado to increase survival in the blood and PMNs *in vitro* and survival and virulence in a mouse model *in vivo*, and may produce dAdo to cause monocytopenia although the activity of Snts to convert dAMP to dAdo has not been experimentally demonstrated.

To date, there were 151 fully sequenced and publicly available *S. suis* strains (contained SC-19), the conservation of 5’-nucleotidases across these strains by BLAST revealed that a substantial identity (over 90%) between SntA with 73.3% (110/150) SntA-associated proteins, between SntB with 96% (144/150) SntB-associated proteins, between SntC with 96.7% (145/150) of SntC-associated proteins, between SntD with 74% (111/150) of SntD-associated proteins (Table S2). Thus, 97.4% (147/151) *S. suis* strains harboured four 5’-nucleotidases, and shared a substantial identity with each associated Snt. In conclusion, *S. suis* 5’-nucleotidase Snts were conserved in *S. suis*.

In conclusion, this study firmly establishes the additive effects of *S. suis* 5’-nucleotidases in generating Ado, contributing significantly to the pathogen’s ability to evade host immune responses, thereby directly affecting its survival, virulence, and host–pathogen interaction dynamics. This research not only advances our understanding of *S. suis* virulence factors but also contributes to the broader field of infectious diseases, providing a foundation for the development of innovative treatments against bacterial pathogens.

## Supplementary Material

Table S1 Primers.docx

Figure S2.tif

Table S2 conserved analysis of Snts across Streptococcus suis-new.xlsx

S1 The ARRIVE Guidelines checklist.pdf

Figure S4.tif

Figure S1.tif

Figure S3.tif

## Data Availability

The data supporting the findings of this study are available at Figshare (https://doi.org/10.6084/m9.figshare.25712313).
