# Supplementary material for: Streptococcus suis 5’-nucleotidases contribute to adenosine-mediated immune evasion and virulence in a mouse model
Source: Virulence. 2024 Sep 16;15(1):2401963. doi: 10.1080/21505594.2024.2401963 (PMC11407386; doi:10.1080/21505594.2024.2401963)
Supplement: Table S1 Primers.docx [file KVIR_A_2401963_SM6960.docx]

**Table S1. Primers used in this study**

| **Primers** | **Description and Sequence (5’ → 3’)** | **Source, reference and Products** |
| --- | --- | --- |
| pET30a-*sntA-F* | cgggatccgaagagattctcaacacaactccagcttct | ORFs of *sntA* genes to construct recombinant prokaryotic plasmids |
| pET30a-*sntA-*R | ccgtcgacatgttcttttttcttagtccatgcgccg |  |
| pET30a-*sntB-F* | cgggatccaccacaaccaatcagccagcaa | ORFs of *sntB* genes to construct recombinant prokaryotic plasmids |
| pET30a-*sntB-R* | ccgctcgagattttcttttctacgacctactgccacgg |  |
| pET30a-*sntC-F* | cgggatccatggatgaattagctgtccaaattatggg | ORFs of *sntC* genes to construct recombinant prokaryotic plasmids |
| pET30a-*sntC-R* | ccgctcgagctccccttccttacgtctcact |  |
| pET30a-*sntD-F* | cggggtaccatggactatacagatattagtattctccat | ORFs of *sntD* genes to construct recombinant prokaryotic plasmids |
| pET30a-*sntD-R* | ccgctcgaggtctccatattgtcttttgaaaaat |  |
| pET30a-*sntA*_H209A_*-F* | cttggcaacgcagaatttaactacggccttgcct | ORFs of *sntA*_H209A_ genes to construct recombinant prokaryotic plasmids |
| pET30a-*sntA*_H209A_*-R* | aattctgcgttgccaagtgtgccgacgtcaaa |  |
| pET30a-*sntB*_H161A_*-F* | ctgttggtaacgcagaatttgactttgggatggatca | ORFs of *sntB*_H161A_ genes to construct recombinant prokaryotic plasmids |
| pET30a-*sntB*_H161A_*-R* | ttctgcgttaccaacagccattgcatcatagc |  |
| pET30a-*sntC*_H132A_*-F* | tggggaacgcagagtttgatgagggacttgatgagt | ORFs of *sntC*_H132A_ genes to construct recombinant prokaryotic plasmids |
| pET30a-*sntC*_H132A_*-R* | aaactctgcgttccccaaagttccgtattcaa |  |
| pET30a-*sntD*_H82A_*-F* | gggcaatgcagagtttgaccacggagatgagttg | ORFs of *sntD*_H82A_ genes to construct recombinant prokaryotic plasmids |
| pET30a-*sntD*_H82A_*-R* | caaactctgcattgcccaaggtcatagcctga |  |
| pET30a-*sntD*_D13A_*-F* | atggactatacagatattagtattctccataccaatgcaatgcattcctacatggagaattttc | ORFs of *sntD*_D13A_ genes to construct recombinant prokaryotic plasmids |
| pET30a-*sntD*_D13A_*-R* | ccgctcgaggtctccatattgtcttttgaaaaat |  |
| pET30a-*sntD*_H15A_*-F* | atggactatacagatattagtattctccataccaatgatatggcatcctacatggagaattttccaaaga | ORFs of *sntD*_H15A_ genes to construct recombinant prokaryotic plasmids |
| pET30a-*sntD*_H15A_*-R* | ccgctcgaggtctccatattgtcttttgaaaaat |  |
| pET30a-*sntD*_G80A_*-F* | gaccttggcaaatcatgagtttgaccacggaga | ORFs of *sntD*_G80A_ genes to construct recombinant prokaryotic plasmids |
| pET30a-*sntD*_G80A_*-R* | catgatttgccaaggtcatagcctgacaacca |  |
| pET30a-*sntD*_N81A_*-F* | ttgggcgcacatgagtttgaccacggagatga | ORFs of *sntD*_N81A_ genes to construct recombinant prokaryotic plasmids |
| pET30a-*sntD*_N81A_*-R* | aactcatgtgcgcccaaggtcatagcctgaca |  |
| pET30a-*sntD*_E83A_*-F* | gggcaatcatgcatttgaccacggagatgagttgc | ORFs of *sntD*_E83A_ genes to construct recombinant prokaryotic plasmids |
| pET30a-*sntD*_E83A_*-R* | caaatgcatgattgcccaaggtcatagcctga |  |
| pET30a-*sntD*_F84A_*-F* | atcatgaggcagaccacggagatgagttgcttag | ORFs of *sntD*_F84A_ genes to construct recombinant prokaryotic plasmids |
| pET30a-*sntD*_F84A_*-R* | gtggtctgcctcatgattgcccaaggtcatag |  |
| pET30a-*sntD*_R336A_*-F* | cggtattgcagcttccttgtcggcgggaccaa | ORFs of *sntD*_R336A_ genes to construct recombinant prokaryotic plasmids |
| pET30a-*sntD*_R336A_*-R* | aggaagctgcaataccgaagccgttgatgacc |  |
| pET30a-*sntD*_F355A_*-F* | gcatccaagcgagtactcttagttcgtatcaa | ORFs of *sntD*_F355A_ genes to construct recombinant prokaryotic plasmids |
| pET30a-*sntD*_F355A_*-R* | agtactcgcttggatgcgggcaagaccttgaccaagtc |  |
| pET30a-*sntD*_F411A_*-F* | ccaattctgcagtttggagcggtaaggacaact | ORFs of *sntD*_F411A_ genes to construct recombinant prokaryotic plasmids |
| pET30a-*sntD*_F411A_*-R* | ccaaactgcagaattggtcacaatctgatagtcctt |  |
| pET30a-*sntA*_A510R_*-F* | gcgatgcttcacgatatacagacattcctgctggccc | ORFs of *sntA*_AYY_ genes to construct recombinant prokaryotic plasmids |
| pET30a-*sntA*_A510R_*-R* | atatcgtgaagcatcgcctcgagtaccagcct |  |
| pET30a-*sntA*_Y530F_*-F* | cctctttgacaacgttgtagccatcttaaaagt |  |
| pET30a-*sntA*_Y530F_*-R* | caacgttgtcaaagaggtaaaggtctgctacgttcttg |  |
| pET30a-*sntA*_Y633F_*-F* | ccaacaactttcgtgcaaacggtactttccct |  |
| pET30a-*sntA*_Y633F_*-R* | tgcacgaaagttgttggtcaccacgatgaatt |  |
| *sntA-*P1 | tgtaagaaaccacaggcaaccg | Fragments produced in *sntA* deletion construction procedure |
| *sntA-*P2 | aacatgtattcacgaacactgaaacgaaaattcataagaatcctccattt |  |
| *sntA-*P3 | tgaattttcgtttcagtgttcgtgaatacatgttataataactataactaataacgtaacgt |  |
| *sntA-*P4 | tattttaatgttcttttttcttagttgtaaaatataacgcccaattaaggtgtgag |  |
| *sntA-*P5 | ccttaattgggcgttatattttacaactaagaaaaaagaacattaaaatagacgagactgagc |  |
| *sntA-*P6 | tgaaacaaagataggcttcctcctagtg |  |
| *sntA-*P7 | ctattttaatgttcttttttcttagtactgaaacgaaaattcataagaatcctccattttc |  |
| *sntA-*P8 | ggattcttatgaattttcgtttcagtactaagaaaaaagaacattaaaatagacgagactgagc |  |
| *sntA-*H209A*-F* | cttggcaacgcagaatttaactacggccttgcct | Site-mutagenesis strain with His-209 in *sntA* to Ala (cac→gca) |
| *sntA-*H209A*-R* | aattctgcgttgccaagtgtgccgacgtcaaa |  |
| *sntB-*P1 | accgtataaaaaacatccagtgagtgtcttg | Fragments produced in *sntB* deletion construction procedure |
| *sntB-*P2 | catgtattcacgaacggtcgtagaaaagaaaattaatcaatcatctgaaaacagg |  |
| *sntB-*P3 | ttcttttctacgaccgttcgtgaatacatgttataataactataactaataacgtaacgtgac |  |
| *sntB-*P4 | aaaaagaaaaaaatctgtaaaatataacgcccaattaaggtgtgagca |  |
| *sntB-*P5 | gcgttatattttacagatttttttctttttcataaatagacctttcttcggcatttt |  |
| *sntB-*P6 | tcatcacccagatgagaaaaatgtagaggacc |  |
| *sntB-*P7 | tctatttatgaaaaagaaaaaaatcggtcgtagaaaagaaaattaatcaatcatctgaaaaca |  |
| *sntB-*P8 | ttgattaattttcttttctacgaccgatttttttctttttcataaatagacctttcttcggca |  |
| *sntB-*H161A*-F* | ctgttggtaacgcagaatttgactttgggatggatca | Site-mutagenesis strain with His-161 in *sntB* to Ala (cat→gca) |
| *sntB-*H161A*-R* | ttctgcgttaccaacagccattgcatcatagc |  |
| *sntC-*P1 | gggctttcttggttgttaataaagaaggc | Fragments produced in *sntC* deletion construction procedure |
| *sntC-*P2 | aacatgtattcacgaacccgaatattctttttcataagcaaaacaatgct |  |
| *sntC-*P3 | tgaaaaagaatattcgggttcgtgaatacatgttataataactataactaataacgtaacgt |  |
| *sntC-*P4 | ccccttccttacgtcttgtaaaatataacgcccaattaaggtgtgag |  |
| *sntC-*P5 | gcgttatattttacaagacgtaaggaaggggagtagga |  |
| *sntC-*P6 | tattgtgccgtatttggaacagcc |  |
| *sntC-*P7 | ccccttccttacgtctccgaatattctttttcataagcaaaacaatgct |  |
| *sntC-*P8 | aaaaagaatattcggagacgtaaggaaggggagtagga |  |
| *sntC-*H132A*-F* | tggggaacgcagagtttgatgagggacttgatgagt | Site-mutagenesis strain with His-132 in *sntC* to Ala (cat→gca) |
| *sntC-*H132A*-R* | aaactctgcgttccccaaagttccgtattcaa |  |
| *sntD-*P1 | gtgttggttacttgggaaccaatactcc | Fragments produced in *sntD* deletion construction procedure |
| *sntD-*P2 | gttattataacatgtattcacgaacactaatatctgtatagtccatactattttttcttgttttttgct |  |
| *sntD-*P3 | gttcgtgaatacatgttataataactataactaataacgtaacgt |  |
| *sntD-*P4 | gtctccatattgtctttttgtaaaatataacgcccaattaaggtgtgag |  |
| *sntD-*P5 | tgggcgttatattttacaaaaagacaatatggagactaaaatggaattaagaagacc |  |
| *sntD-*P6 | atctttctactccgtctcgaacatctt |  |
| *sntD-*P7 | cattttagtctccatattgtcttttactaatatctgtatagtccatactattttttcttgttttttgct |  |
| *sntD-*P8 | agtatggactatacagatattagtaaaagacaatatggagactaaaatggaattaagaagacc |  |
| *sntD-*H82A*-F* | gggcaatgcagagtttgaccacggagatgagttg | Site-mutagenesis strain with His-82 in *sntD* to Ala (cat→gca) |
| *sntD-*H82A*-R* | caaactctgcattgcccaaggtcatagcctga |  |
| pSET2-*sntB-F* | cgggatccgaaatgagcggattagatagctaattcgc | To build complemented strain of *ΔsntB* |
| pSET2-*sntB-R* | cggaattcttaattttcttttctacgacctactgccac |  |
| pSET2-*sntC-F* | cgggatccgaacagttctcttccgtttggaagaga | To build complemented strain of *ΔsntC* |
| pSET2-*sntC-R* | tcccccgggctactccccttccttacgtctcact |  |
| pSET2-*sntD-F* | aactgcagtgaagcaggataaaaaagcgcg | To build complemented strain of *ΔsntD* |
| pSET2-*sntD-R* | cggaattcttagtctccatattgtcttttgaaaaattcactgatga |  |
| *sntA*-in-F | ggtctagcaaaaacagctgttcttatcga | Truncated ORF of *sntA* gene |
| *sntA*-in-R | agataggattggtagattagcttctgaagtacc |  |
| *sntA*-out-F | agcctccatatcatccaaagttacatcc | ORF of *sntA* and its upstream and downstream arms |
| *sntA*-out-R | gtaggatgaagggtgtttttggcgt |  |
| *sntB*-in-F | aacacgttcttctacaggaagagttggatc | Truncated ORF of *sntB* gene |
| *sntB*-in-R | acaaggaaaccttgaacttcccgct |  |
| *sntB*-out-F | ttgaataacctcttcaagggctggataatag | ORF of *sntB* and its upstream and downstream arms |
| *sntB*-out-R | gggttgaaatctggtgttatctctgtcat |  |
| *sntC*-in-F | acagctttaaagtcggcaatatctgtatca | Truncated ORF of *sntC* gene |
| *sntC*-in-R | ttttagcagatgaattagctgtccaaattatggg |  |
| *sntC*-out-F | ctgttttagcaggattactaggtctaccaact | ORF of *sntC* and its upstream and downstream arms |
| *sntC*-out-R | ttcagcagaattggcagcagatatt |  |
| *sntD*-in-F | tgtccttaccgctccaaacaaaaga | Truncated ORF of *sntD* gene |
| *sntD*-in-R | atcgagctgatgaatcaaattggttgtcag |  |
| *sntD*-out-F | tttcctgttttgattcagcagtttgtcact | ORF of *sntD* and its upstream and downstream arms |
| *sntD*-out-R | acaaaatccaaaggctttggaagctg |  |
