# Supplementary figures and images for: Streptococcus suis 5’-nucleotidases contribute to adenosine-mediated immune evasion and virulence in a mouse model
Source: Virulence. 2024 Sep 16;15(1):2401963. doi: 10.1080/21505594.2024.2401963 (PMC11407386; doi:10.1080/21505594.2024.2401963)

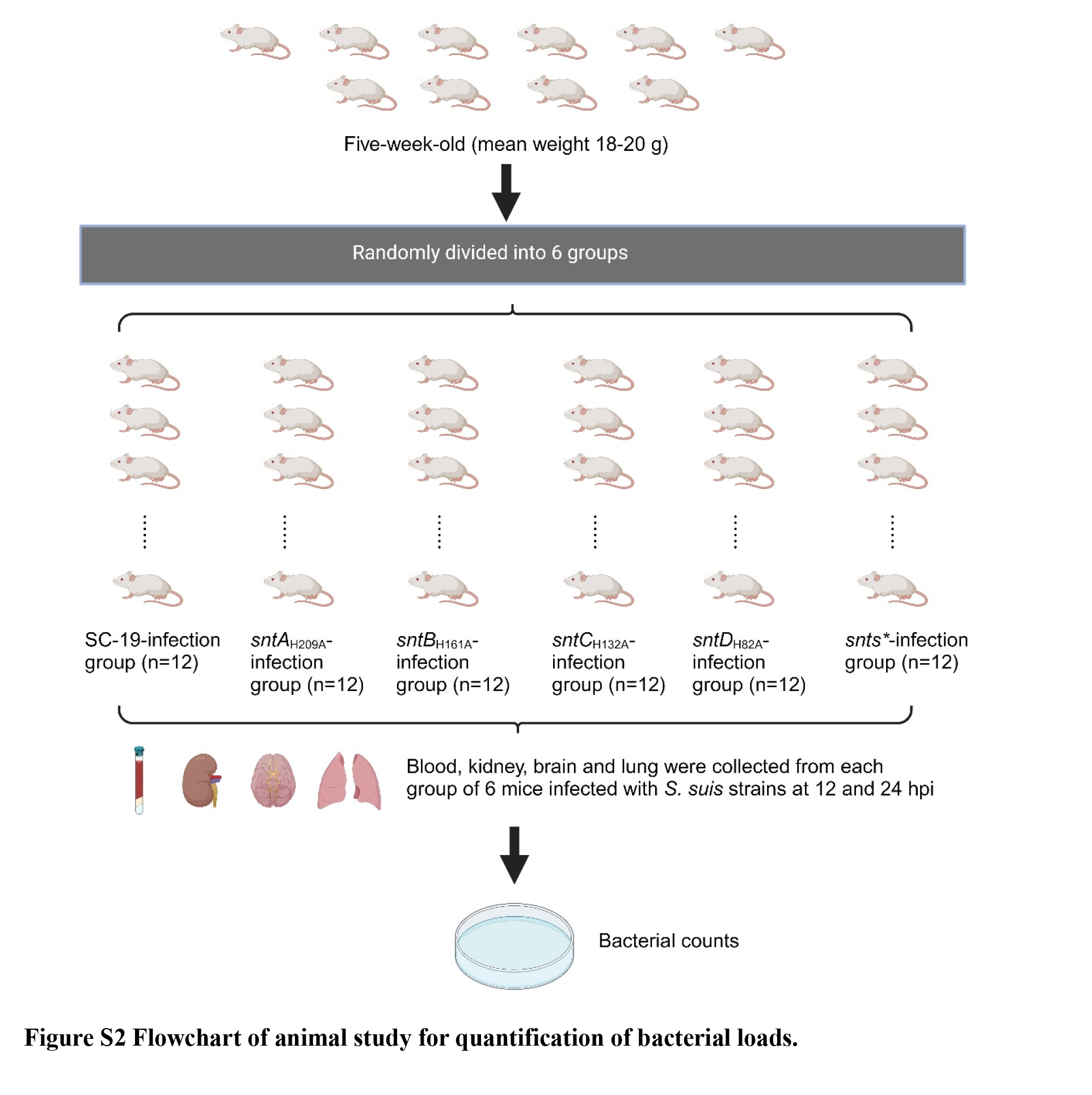

Supplement: Figure S2.tif [file KVIR_A_2401963_SM6959.tif]

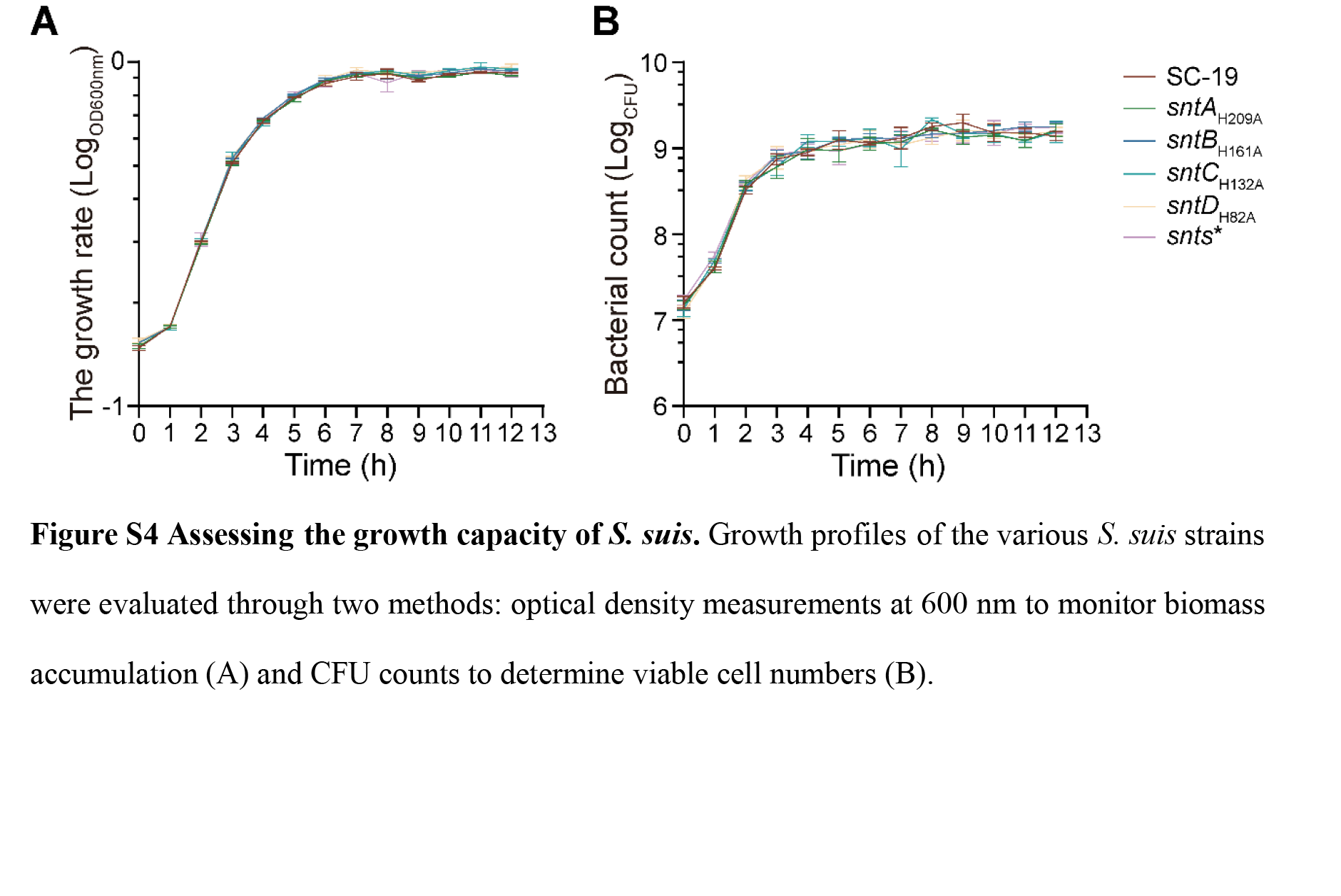

Supplement: Figure S4.tif [file KVIR_A_2401963_SM6956.tif]

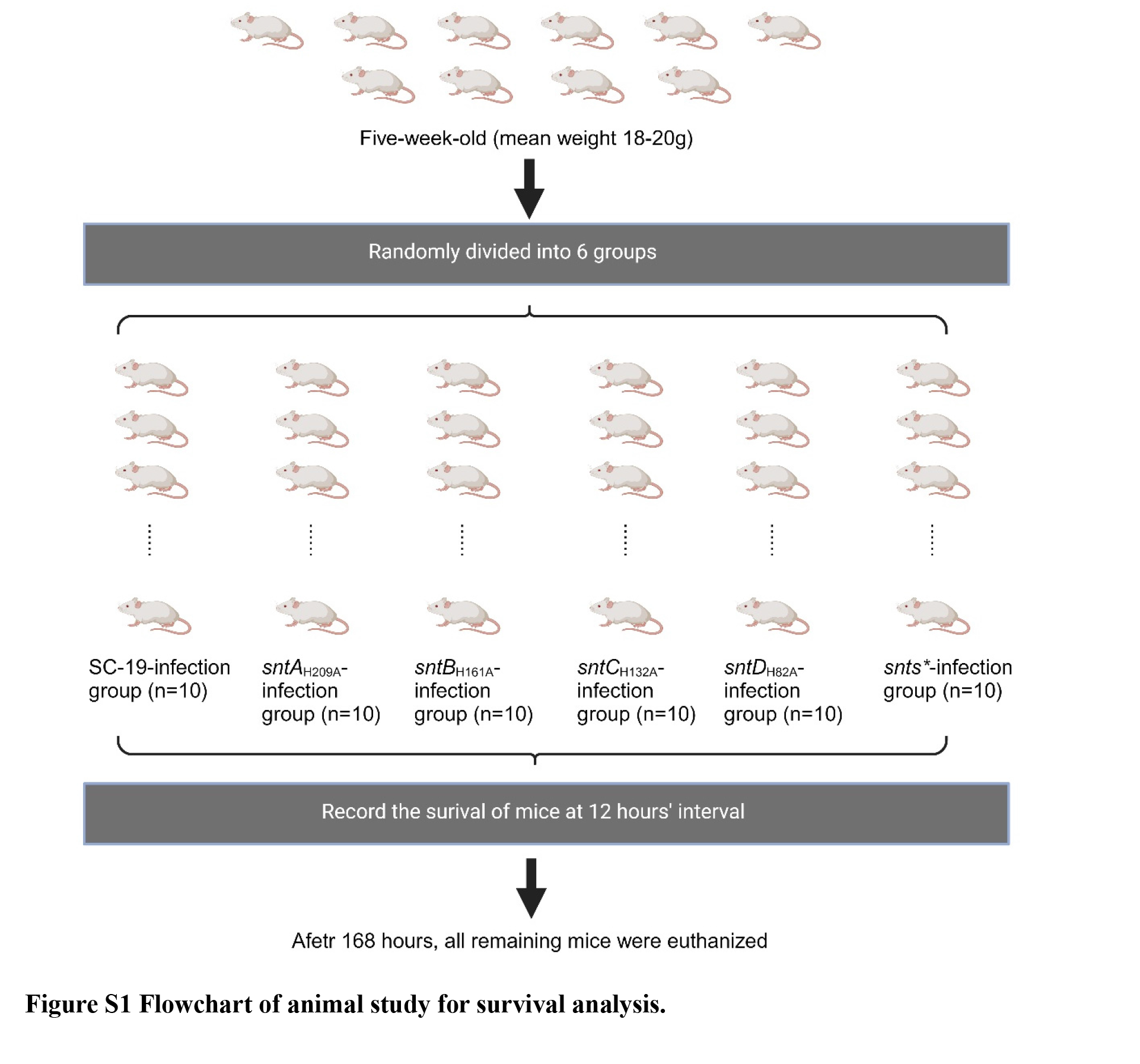

Supplement: Figure S1.tif [file KVIR_A_2401963_SM6955.tif]

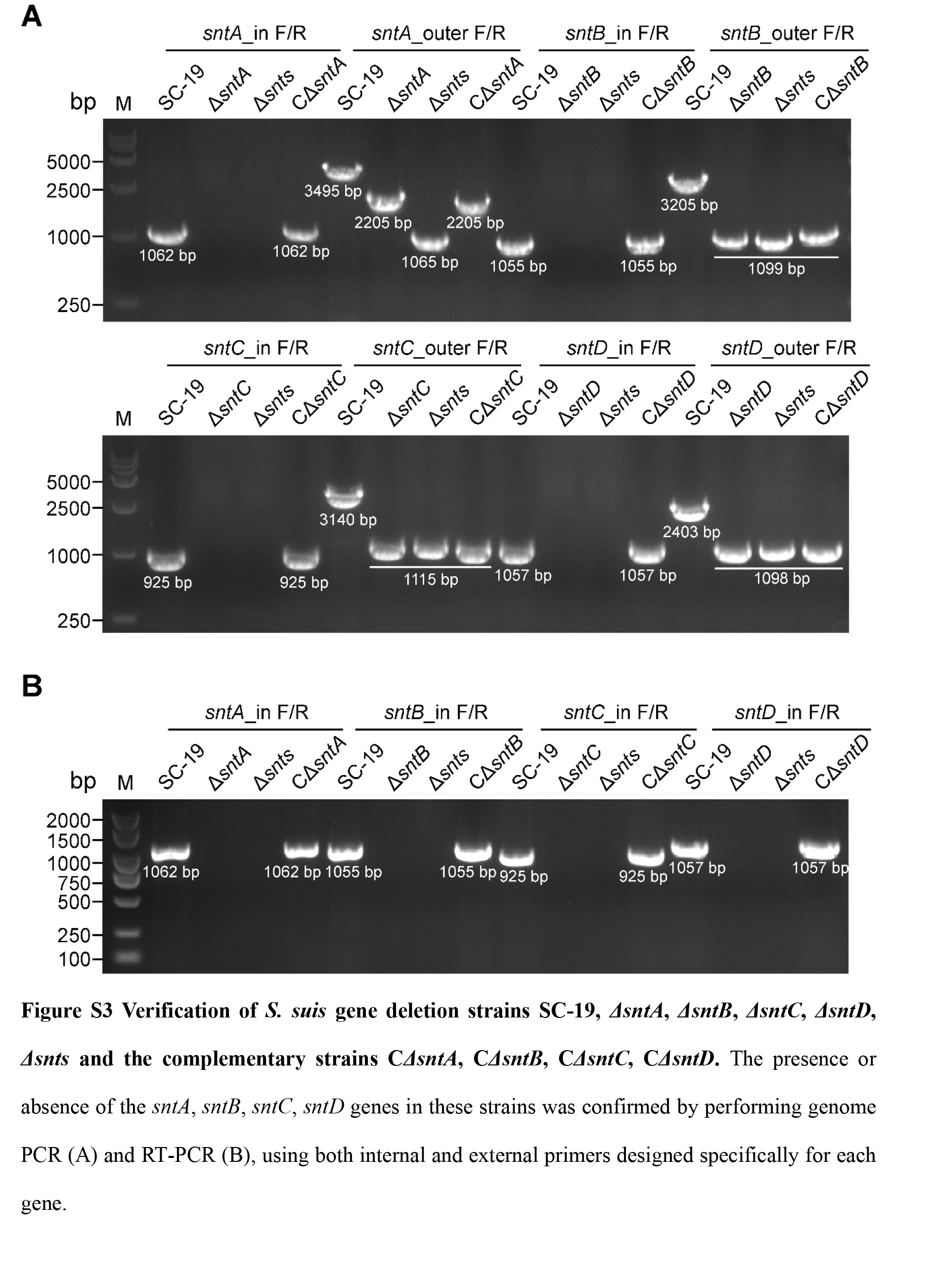

Supplement: Figure S3.tif [file KVIR_A_2401963_SM6954.tif]
